# Review of Luminescence-Based Light Spectrum Modifications Methods and Materials for Photovoltaics Applications

**DOI:** 10.3390/ma16083112

**Published:** 2023-04-15

**Authors:** Maciej Sibiński

**Affiliations:** Department of Semiconductor and Optoelectronic Devices, Lodz University of Technology, al. Politechniki 10, 93-590 Lodz, Poland; maciej.sibinski@p.lodz.pl

**Keywords:** photovoltaics, luminescence, light shifting, light conversion, flat concentrators

## Abstract

The dynamic development of photovoltaic and photo-sensitive electronic devices is constantly stimulated by material and technological advances. One of the key concepts that is highly recommended for the enhancement of these device parameters is the modification of the insulation spectrum. Practical implementation of this idea, although difficult, may be highly beneficial for photoconversion efficiency, photosensitivity range extension, and their cost reduction. The article presents a wide range of practical experiments leading to the manufacturing of functional photoconverting layers, dedicated to low-cost and wide-scale deposition methods. Various active agents, based on different luminescence effects as well as the possible organic carrier matrixes, substrate preparation and treatment procedures, are presented. New innovative materials, based on their quantum effects, are examined. The obtained results are discussed in terms of the application in new generation photovoltaics and other optoelectronic elements.

## 1. Luminescence Mechanisms and Their Utilization in Photovoltaics

The luminescence phenomenon is described as the emission of electromagnetic radiation in excess of thermal radiation. In most cases, the emitted radiation occurs within the visible range, even though ultraviolet emission and infrared emission were also observed [1]. Luminescence is traditionally divided according to the excitation source type [2], which allows for the definition of photoluminescence, electroluminescence, cathodoluminescence, X-ray luminescence, triboluminescence, and chemiluminescence, with the most prominent role belonging to the first two mechanisms.

Luminescence is also often characterized by the time of extinction, measured as the effect duration after the disappearance of the excitation. This value may vary according to the effect mechanism and material construction from 0 to several hours, which provides a hint as to the traditional classification of the phosphorescence (with no afterglow effect) and fluorescence (with notable afterglow) [3]. The role of this effect may be notable in particular applications, as well as during emission intensity measurement processes. Luminescence is also practically used in many applications, including light-emitting diodes (LEDs), storage phosphors, persistent luminescence phosphors, scintillators, and notably in up and down conversion mechanism materials which are designed for photovoltaics [4].

There are many factors causing serious limitations in the actual efficiency of the photovoltaic structures. Amongst them one can distinguish thermalization losses (close to 33% of the total losses), the too small energy of absorbed photons (about 15% of the total loss) or the waste of ultraviolet (UV) light high energy photons and recombination losses (around 15% of total loss) [5,6]. According to the Shockey-Queisser limit [7] the maximum efficiency of a standard crystalline Si solar cell is limited to 31%, which currently leaves only about a 4.3% gap for future improvement [8]. Many concepts have been developed to overcome this constraint, including the following: multijunction or tandem solar cells [9], the implementation of the intermediate band [10], hot carrier [11] and carrier multiplication [12], spectrum split systems [13], or insulation spectrum modifications [14,15].

**Figure 1:**
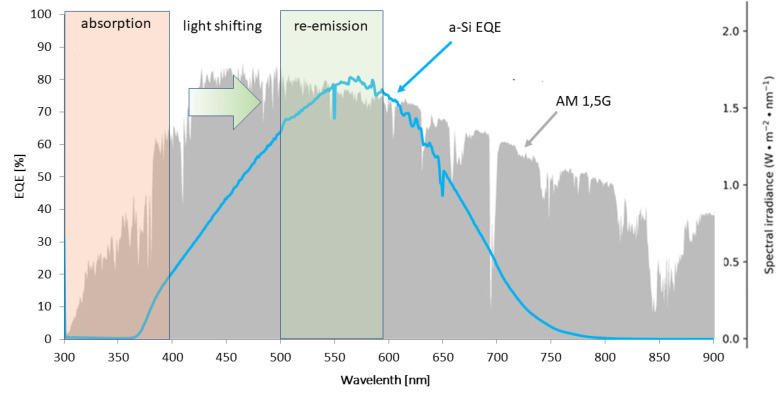
General idea of down-shifting process flow in an amorphous silicon solar cell. The ultraviolet range of the solar irradiation spectrum is not used by a silicon solar cell efficiently, hence the DS layer is shifting radiation to the range, which is effectively matched to a specific solar cell quantum efficiency [22].

The mechanisms of these spectral modifications may fall into one of three main categories: up-conversion (UC), based on the absorption of two low-energy photons and the subsequent emission of one higher energy photon; down conversion (DC), where one high energy photon is absorbed and converted into two lower-energy ones; and down shifting (DS), with the conversion of one high energy photon into one with lower energy and an additional energy emission [9,10,11,12,13,14,15]. Up conversion, as the non-linear process with low probability, did not find many practical applications in photovoltaics due to their low efficiency and natural limited amount of energy in the infrared range of the light spectrum. Consequently, the DC process is interesting, but is also limited, as it is necessary to introduce an intermediate level precisely in the center of the semiconductor bandgap and eliminate losses to generate two photons from the single absorbed one. Finally, the last of these processes was practically proposed in a PV application by Hovel et. al. in 1979 [16]; however, the general concept was formulated a few years before as a solar luminescent concentrator [17,18]. It is estimated that this solution may lead to even 10% efficiency growth in solar cell structures when employed properly [19,20]; however, the current progress in cell parameters is still lower [21]. The schematic idea of the process aim is shown in Figure 1, where the EQE characteristics of the a-Si cell is shown in the perspective of an AM 1.5 spectrum. UV photons are subsequently converted into visible light, and are thus matched with the photoconversion range of a specific cell type.

**Figure 2:**
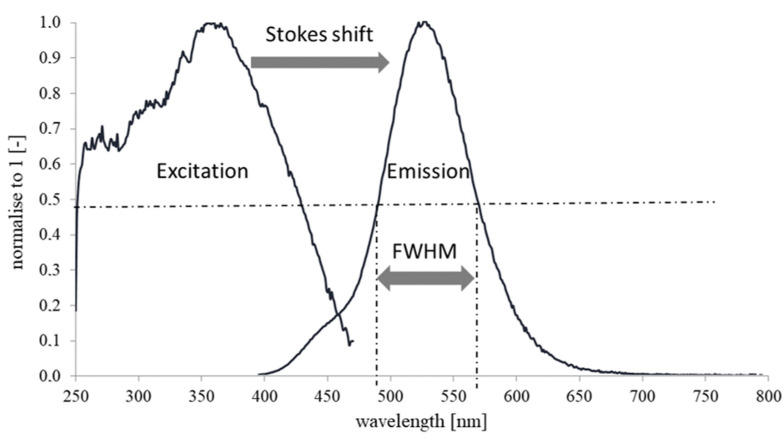
Explanation of the most important optical parameters in the UV to visible light conversion process.

The critical parameters of the process are connected with the illumination and excitation characteristics. The specific distance between the excitation and emission peaks is called the Stokes Shift, and the peak position and the curve dissipation of the emission part may be properly characterised by the full width at half maximum (FWHM) parameter. A proper Stokes shift prevents the overlapping of the excitation and emission regions, whereas the FWHM value shows the average dissipation width of a specific spectrum, which is shown in Figure 2. For the best performance of light shifting effect peak emission, the emission spectrum of the FWHM should be close to the maximum value of specific solar cell EQE characteristics.

**Figure 3:**
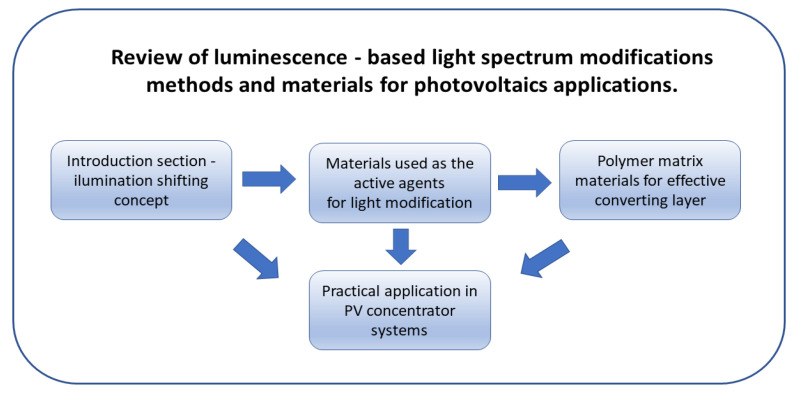
Schematic of the presented article scope and flow.

In either case, the most important issue in achieving these goals is the finding and suitable processing of the proper material candidate for effective spectrum modification. Detailed investigations of the possible candidates for this role is a subject of the following sub-chapters. Consequently, the effective converting layer preparation may only be accomplished when the effective carrier matrix that is appropriate for this role is prepared. This will be explained in Section 2. Section 3 is dedicated to the practical constructions and applications of possible converters. The schematic of the presented review is briefly depicted in Figure 3.

### 1.1. Down Conversion by Metal Oxides-ZnO Nanoparticles

Metal oxides (MO) are potential materials for several applications in electronics, due to their long term stability, possible wide bandgap range of 1–10 eV, relatively low price, and nontoxix character [23]. Additionally, many of them may be processed as semi-transparent layers, depending on the technological treatment. In the field of optoelectronics, MO in the form of thin films or bulk were used in laser diodes, light emitting diodes (LED) [24], photodetectors [25] and solar cells [26]. Their prominent role in these applications fulfilled transparent conductive layers (TCO’s) used in the form of front contact for many popular solar cell structures such as CIGC, CdTe/CdS, dye-sensitized (DSSC), or organic solar cells [27,28]. For this role, many variants of MO were proposed, with the most popular being high transparent and low resistance indium-tin oxide (ITO) [29].

With some of these materials it is also possible to achieve the nanoscale particles (NPs) with the effective bandgap increased in comparison to the bulk material. This method is the blueshift of light absorption, and effective emission may also be achieved. The highest efficiency of this process takes place when the highest density of states is achieved [30]. These structures very often present a wide absorption range, high emission intensity, and good photostability. Amongst these materials, many types of NPs were fabricated with different particle parameters. A short summary of these methods and the employed oxide materials is presented in Table 1.

ZnO is a popular II–VI group material with a direct wide band gap, equal to 3.37 eV at room temperature, and semiconducting properties [39]. Due to the relatively low cost, bio-compatibility, and high accessibility, it has been used in several instances, including optical applications in the UV spectral range [40,41]. It also has been widely used historically in photovoltaic applications in the form of ZnO:Al (AZO) layers, which are applied as transparent contact materials in CIS/CIGS [42], and in organic [43] and hybrid solar cells [44]. Aluminum doped ZnO layers have also been used as transparent electrodes in the silicon cells [45], as well as in antireflective coatings by the creation of a refractive index matching layer [46].

ZnO is characterized by a higher absorption coefficient than other wide bandgap materials such as GaN, but the proper crystalline quality of the material is required for the high efficiency and high sensitivity of ZnO based devices in the UV region, since the recombination on intrinsic defects deteriorates the overall efficiency dramatically. Thus, many research groups put an initially strong effort into the elimination or reduction of these defects [47,48]. Gradually, it appeared that these defects might result in light emissions within a relatively wide range from the visible region to IR [49,50]. This phenomenon was observed prominently in nanostructures, which was also a subject of investigation of a study [51]. It was stated that the high concentration of defects may be achieved in nanostructures due to their normal high surface to volume ratio. Consequently, this material may be a promising candidate for white light emitters [52], photoreactive layers under visible light illumination [53], and particularly energy down-shifting systems in photovoltaics [54]. Various methods of ZnO nanostructure synthesis were proposed, including ultrasonic synthesis [55], low energy cluster beam deposition (LECBD) [56], the hydrothermal method [57], and lastly the sol-gel synthesis method [58,59]. It was stated that several parameters influence the final luminescence intensity. One of them is the optimal nanoparticle diameter, which should lay within the range of 100–200 nm.

However, a lot of improvements are still expected to be completed, since the synthesis is difficult to control, and mechanisms of defect emission are controversial [60,61]. It is also believed that the proper matrix structure and layer deposition technique is critical for the final ZnO NPs layer performance. So far the efficiency progress of solar cells with the implemented ZnO NPs-based nanoconverters did not exceed a fraction of a percent either in simulations or in experiments [53].

### 1.2. Rare Earth Elements and Other Inorganic Materials for Luminescent Spectrum Modifications

There are a variety of inorganic compounds used for the achievement of a luminescent effect, but most are connected with a few amin groups. First and arguably the most popular is the one based on rare-earth elements. These fifteen elements belong to the lanthanides (marked Ln), having atomic numbers ranging from 57–71 and being characterized by well-separated energy level structures, which provides the possibility of radiative emission [62]. Additionally, two elements apart from the lanthanides group (scandium and yttrium) are also accounted for in this set. Following their name, there is lack of ubiquitous access to these materials, which causes production problems [63], but their unique parameters explain their profound exploration.

**Figure 4:**
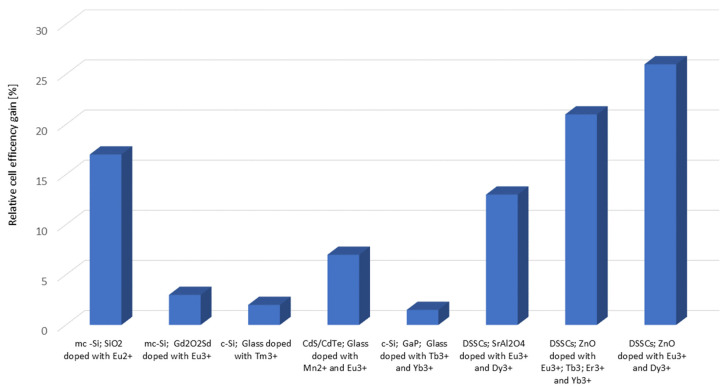
Relative efficiency of various solar cell categories, equipped with specific converting layers, based on different rare earth elements, based on [22,67,68,69,70,71].

For the photoluminescence effect of these materials, the most important are 4f-5d interband transitions, which are responsible for the UV emission and 4f-4f interband transitions, connected with the visible range [64]. An additional advantage, important for the practical use of these materials, is their low interaction with the chemical environment, which is caused by the shielding of the 4f orbitals by 5p and 5s shells [65]. Both luminescence and phosphorescence effects with an afterglow of up to several hours were obtained this way. Thus, the chemical compounds with the rare-earth elements are often investigated with regard to their implementations in spectrum-shifting PV applications. In these applications, they are characterized by a high luminescence efficiency and a wide absorption spectrum, but are often jeopardized by low absorption coefficient values [66]. This problem is often addressed by the increasing of the layer thickness and the manipulation of the layer composition; however, the trade-off between the optical transmission in the visible region and the luminescence process effectiveness is always an issue. Nevertheless, several experiments were undertaken to implement rare-earth elements in photovoltaics. The most important achievements for each type of material, implemented in specific solar cell constructions, are presented in Figure 4.

**Figure 5:**
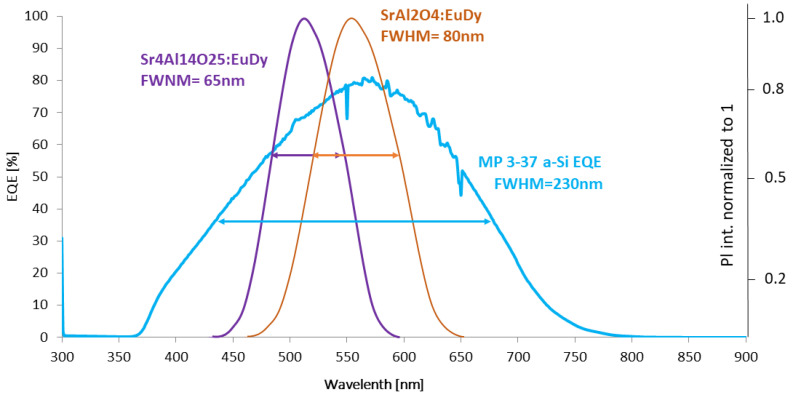
EQE characteristics of commercially available, flexible a-Si solar cells (MP3–37) and luminescent layer emission spectrums normalized to 1 for Sr_4_Al_14_O_25_: Eu, Dy and SrAl_2_O_4_: Eu, and Dy with FWHM of each characteristic [77].

The extensive research conducted on this topic [72,73] has led to the conclusion that under some conditions these materials may be effectively used for the adjusting of excitation-emission spectra according to several PV device demands, including popular Si cells. Some promising materials based on Eu and Dy were used by several groups. They provided the possibility of efficiency improvement in various cells’ structures and configurations, most prominently for quantum dot-sensitized solar cells (QDSSCs) [74,75]. These materials even became commercially available in the form of luminescent powders such as Sr_4_Al_14_O_25_: Eu, Dy (BGL-300M), and SrAl_2_O_4_: Eu, and Dy (G-300M) by NEMOTO LUMIN-NOVA^®^ [76]. Figure 5 shows the adjustment of the emission spectra for two compositions: pigments Sr_4_Al_14_O_25_: Eu, Dy, and SrAl_2_O_4_: Eu, Dy in a polymer base according to a-Si cell demands.

Apart from rare-earth elements, some other inorganic compounds are also tested for illumination modifications in PV devices. One of the verified candidates was calcium copper tetrasilicate, CaCuSi_4_O_10_, [78] which is also widely known as Egyptian Blue, due to its long history as an ornamental ink in ancient Egypt. This material is available naturally in the form of a cuprorivaite mineral, but may also be synthesized during the calcination of silicon in the sintering process in the temperature range of 850–1000 °C. It is characterized by the clear excitation and definition of emission curves and a beneficial Stokes shift greater than 280 nm. Furthermore, the long-term stability is not an issue, since some active layers are still preserved from the time of ancient Egypt. The challenges, however, are mostly connected with the excitation peak position within the visible range (610 nm) and the proper selection of the matrix material. Due to these issues, so far there are no examples of operating convertors based on Egyptian Blue for solar cell applications. However, such a system is still possible, and the specific conditions for the effective implementation with reliable luminophore photostability were formulated in the literature [79].

### 1.3. Organic Luminophores

Organic materials and organic dyes are growing rapidly, finding their applications in solar cells [80,81], photo detectors [82], and also in the luminescent applications with visible and infrared emissions [83]. Their inherent advantages, connected with their high availability, low energy consumption production processes, and flexibility, as well as many possible compositions and deposition techniques, make them attractive candidates for these applications, but many obstacles have yet to be solved for efficient and stable device production.

**Figure 6:**
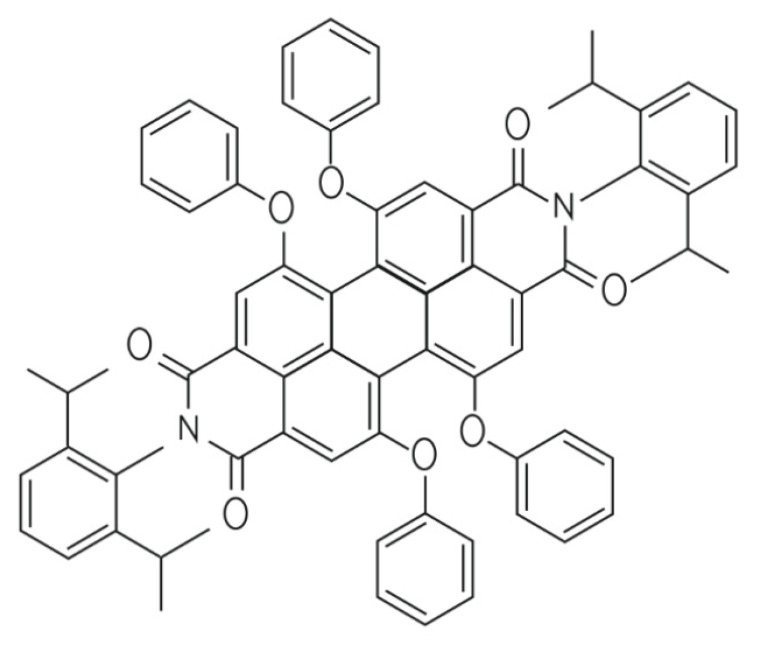
Structure of C_72_H_58_N_2_O_8_ (N, N′-bis (2,6-di-isopropylphenyl) organic luminophore.

Various materials were used so far for non-metallic luminescent layer production. They may be divided into six main groups according to their structural construction: difluoroboron diaroylmethanes, diarylketones, diarylsulfones, triazines and pyrimidines, fused phenazines, and N-arylcarbazoles [84]. Although all of the mentioned structures are promising photoluminescence and phosphorescence materials, their applications in functional layers and devices remains a challenge. Recently successful experiments on fully organic Perylene Red with the formula C_72_H_58_N_2_O_8_ (N, N′-bis (2,6-di-isopropylphenyl) for luminescent solar concentrator (LSC) were reported [85]. The general structure of this material is presented in Figure 6.

This material, which is based on polycyclic aromatic hydrocarbon, is characterized by high stability, high, close to unity quantum yield, and hydrophobic properties, which led to the practical experiments in luminescent concentrators [86,87]. Unfortunately, the low value of the Stokes shift, which is only 35 nm, accompanied by the relatively strong visible absorption significantly limited the actual efficiency of the modified solar cells. Additional issues were related to the proper carrier matrix composition for luminescence effect preservation, and typically for the organic optical layers with long-term stability.

Lastly, research on chlorophyll (Chl) was also conducted with regard to this application. Initially, Chl, being the fundamental compound of photochemical conversion in plants, was used in photovoltaics, mainly as the dye material in dye-sensitized solar cells [88]. It gradually appeared that the application of this layer may lead to a broadening of the absorption spectrum for hybrid and organic cells [89,90]. This directly led to the idea of the implementation of a Chl converting layer for inorganic solar cells as the down-shifting material [91].

Based on this observation, some technological work was undertaken. Simple preparation methods, based on polyvinyl alcohol (PVA) and aliphatic waterborne polyurethane (WPU) aqueous solution with an admixture of Chl, were employed. An important factor of this research was the addition of nano SiO_2_ dispersion for the protection of the organic compound against environmental influence [92]. On this basis, the noticeable down conversion effect on organic, dye-sensitized and even Si solar cells were achieved. The obtained Stokes Shifts were higher than 200 nm; however, the FWHM values were rather small. An additional disadvantage is the position of the excitation spectrum peak at a wavelength range of 400–450 nm, which is the place of the natural high quantum efficiency of many solar cells. Moreover, the fast degradation of the fluorescence effect was observed in time, despite the application of SiO_2_.

### 1.4. Quantum Dots and Perovskites in Luminescent Applications

Quantum dot (QDs) materials are widely known, and are often referred to as suitable candidates for light conversion applications typical from the UV to the visible region [93], and also in photodetectors [94,95] Owing to the high density of states, their potential conversion efficiency may surpass the values of other materials [30]. The most notable advantages of QDs are based on their high absorption coefficient and high probability of radiative emission, mainly resulting from the existence of intraband discrete energy levels. This phenomenon is possible due to their quantum confinement, and results in a narrow emission spectrum and a very high luminescence efficiency, which approaches 100%. Additionally, by the introduction of QDs it is possible to provoke an efficient luminescent effect in the materials which have not been luminescent in their bulk forms, such as silicon or carbon.

This is practically difficult due to the challenges in obtaining a proper, cheap and repeatable synthesis process, as well as the deposition and layer formation steps. Nevertheless, they offer some additional assets in the form of narrow emission spectra, which may be adjusted to the absorption properties of a solar cell by the manipulation of nanoparticle sizes.

**Figure 7:**
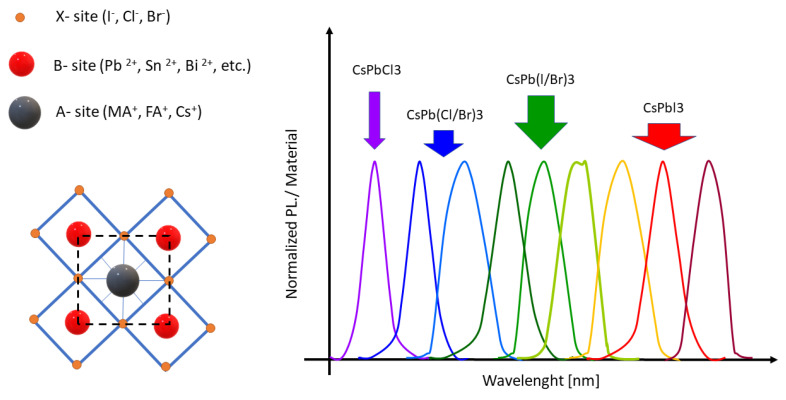
Emission ranges of various material QDs under UV excitation, based on [99] and typical metal halide perovskite construction.

The most popular QDs materials that are used for optical applications are based on cadmium or, more recently, on lead [96] due to its ease of synthesis and suitable optical properties; however, their health hazard properties of cadmium lead to some commercialization problems. Also, the group of QDs based on Pb, such as CsPbBr_3_ QDs in in CsBr:Pb single crystal [97], or CsPbCl_3_-like quantum dots in CsCl: Pb crystals [98], as shown in Figure 7, seems to be controversial in spite of their potential advantages.

This problem also affects all-inorganic lead halide perovskite QDs (IPSK QDs), which have presented a high potential for wavelength-shifters owing to their controllable and high-intensity photoluminescence, high optical absorption coefficients [100], satisfactory chemical stability, and single-component ultra-fast decay time [101]. Despite their successful applications in photovoltaics [102,103], their toxicity jeopardized the wider acceptance of these materials.

In response to these challenges, Pb free perovskites are currently being investigated. The initial approach to this problem was connected with the replacement of lead by tin in the material’s structure, but the high intrinsic defect density and oxidation vulnerability of Sn typically leads to unsatisfactory product properties. Alternatively, Bi^3+^ as the isoelectronic to Pb^2+^, which is a stable and non-toxic material, was tested in this role [104]. Using this method, photoluminescence at the peak from 360 nm to 540 nm was observed, however, the low quantum yield (up to 12%) and material availability are still the issue that is obstructing its successful commercialization.

Apart from this, the most promising heavy-metal free quantum dots are based on ZnS, ZnSe, InP, Si, CuInS2, and recently graphene. This material became very popular owing to its unique mechanical and electrical properties; however, in this particular application, the most important factors are its high abundance, non-toxicity, and possibility for bandgap tailoring, which may result in emission character adjustment. Achieved efficiencies of luminescence are 70% for ZnSe QDs [105], 94% for carbon QDs [106], and up to 81% for ZnCuInS/ZnS core-shell QDs [107], with an emission peak that is adjustable in the range of 50 nm to 610 nm.

## 2. Carrier Matrix Material and Converting Layer Preparation

A critical step that is necessary for functional layer deposition is the preparation of a proper carrier matrix that is suitable for luminescent layer deposition. Finding a suitable material and the preparation process of the matrix is as important for the light shifting layer functioning as the processing of the active luminescent agents. The main features demanded from this kind of layer are as follows:

- The good optical transparency in the UV-Vis spectrum;
- The appropriate refractive index for incoming light reflection reduction and total internal reflection creation;
- An easy method of luminescent compound dispersion;
- The non-suppressive character of the luminescent compound emission effect;
- The strong adhesion to dedicated substrates;
- The possibility of a flexible structure;
- The cheap and scalable method of deposition.

The main groups fulfilling the high transparency demand in the UV-Vis region are quartz, special glass (like borosilicate glass), and some polymers. In typical rigid solar cells, the construction of a highly transparent and durable glass is used, but considering the following mentioned demands, the natural choice of the matrix material will fall on organic materials. Among them, the most popular are acrylic polymers, cyclo-olefin polymers, and silicone polymers [108]. Table 2 presents the basic parameters of these groups.

Amongst this group, the most popular materials are Poly(methyl methacrylate) (PMMA), Polydimethylsiloxane (PDMS), and Cyclic Olefin Polymer (COP). PMMA, being an extremely popular organic acrylic material that is used for car windows, smartphones, aquariums and other transparent applications, was initially the natural choice in many practical experiments of solar spectrum modifications [14,111]. Additionally, the well-developed production technology and relatively low price are important advantages of the material in solar concentrator applications; however, the limited transparency in the UV range may pose a threat to the high energy gain. Furthermore, the low allowable temperature (typically below 100 °C) limits the options for technology processing. This has recently led to the search for new types of carriers in luminescent layers.

PDMS is an inorganic polymer from the silicone family with high flexibility and very low wetting, and thus high humidity resistance. These features, combined with its relatively high temperature resistance, make it attractive for some photovoltaic technological experiments, including the optical enhancement of solar cell efficiency by a special anti-reflection pattern [112]. Future applications of this material, including wavelength shifting layers, remain to be considered, however.

New potentially attractive solutions are based on COP materials that are constructed from cyclic olefin monomers and ethene. The main advantages of these materials are their high strength and robustness, low water absorbance, higher than PMMA temperature endurance, and, most importantly, their higher optical transparency in the UV-Vis range. There are various types of this material with different product names like ZEONEX^®^, TOPAS^®^ or ARTON^®^. Their full potential shall be explored, but some of them have been successfully implemented in wavelength-shifting layers experiments [113].

**Figure 8:**
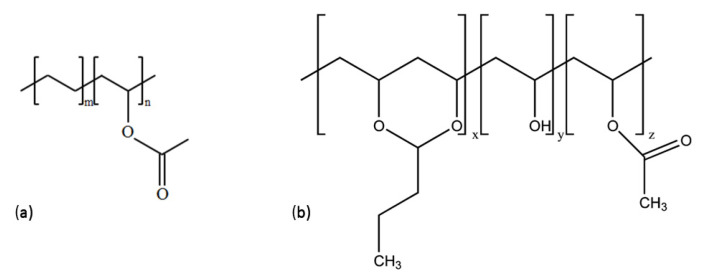
**S**tructure of EVA (**a**) and PVB (**b**) materials.

Alternatively, instead of incorporating an additional wavelength-shifting layer, the concept of standard lamination foil employment as a carrier matrix was proposed [85]. For this purpose, the most frequently used materials are ethylene-vinyl acetate (EVA) or Polyvinyl butyral foil (PVB). The structures of both of these materials are presented in Figure 8.

EVA, being a popular hermitization material for Si solar cells, is also characterized by a high electric resistivity of 10 × 10^12^ Ω·m and a thermal conductivity of k = 0.23 W·m^−1^·K^−1^, as well as a high optical transparency and a refractive index of n = 1.49 [114]. This, together with its popularity and well-known production process, makes it a good candidate for this role, especially when organic agents are used. Unfortunately, it appeared that the utilization of EVA as the host matrix did not provide satisfactory photostability for organic luminescent agents, and presents limited solubility [115]. Instead, PVB proved to have more potential in this role. This material was previously used in safety glass, with the foil thickness varying typically from 50 µm to 250 µm, the temperature melting point from 100 °C to 190 °C, and having a high optical transparency and refractive index of 1.48. PVB is a resin that is soluble in organic solvents, especially alcohol, but not in hydrocarbons, and is resistant to acids and alkali. Additionally, the final product is solvent and dust free, and no migration of additives or plasticizers from the material was observed. Recently, this material has also been used for PV cells’ hermitization [116]. Some experiments aimed at the application of organic luminophores directly into the PVB matrix were performed successfully [85]. The photostability results of organic luminophore during several thousands of hours under AM 1.5 illumination was achieved, and the overall efficiency gain was 0.5%.

There are several technologies used for the preparation of the host matrices currently under investigation, depending on the type of the luminophore and the matrix material type. The most frequently used depend on the mechanical mixing of luminescent agent powder with the polymer base by stirring. Sometimes additional active substances such as alcohols are added for better solubility of the powder in the specified matrix. The most popular methods of layer deposition used for experiments with luminescent materials in a polymer host matrix are:

- Dip coating;
- Spin coating;
- Spray coating;
- Printing technologies.

All mentioned techniques are applicable for liquid materials. Spin coating is used for the deposition of the uniform layer by the high speed rotation of a liquid substance on the circular substrate, with the speed varying from a few hundred to a few thousand rpm [117]. With this technology, the uniform layers with a thickness varying from nanometers to several micrometers may be achieved for different materials. Due to these features, spin coating is currently used in semiconductor processing technologies [118,119]. This method is relatively fast, inexpensive, and repeatable, however possess some obvious shortcomings. The first is connected with the limited area and the defined shape of the potential substrate. Second, also observed in the experiments was the high centrifugal force, which may result in the non-uniform distribution of the luminescent particles in the polymer host matrix after the stirring process.

During spray coating, the ink material is atomized into a special spray nozzle, forming an aerosol according to the parameters of the ink parameters as well as the gas type and pressure, humidity, and temperature [120]. Additional important factors are the distance between the nozzle and the substrate, the deposition time, and the nozzle diameter. In the professional production process, all mentioned factors are computer-controlled, and the process is done fully automatically, which results in fine parameter regulation [121]. This also creates a great opportunity for the preservation of the uniform layer microstructure and high coating bond strength, which combined with high speed and unlimited surface area and shape creates the best chance for luminescent layer mass production.

Dip coating is a laboratory and industrial method used for the production of many layers, including popular polymer ones such as PMMA and PEDOT–PSSS [122]. It is a scalable process, which is a waste-free method offering a good control of the deposited layer thickness. During this process, the sample is immersed in the ink container for a specified period of time and is subsequently pulled off for the ink evaporation and solid layer formation, often in an environment of increased temperature and a neutral atmosphere. The process takes place on both sides of the material, which makes it effective for the hermitization of flexible layers, but not suitable for the creation of light converting the front covering without any additional protection.

Printing methods cover many specific variants of layer deposition, including screen-printing, rotogravure, ink-jet printing, blade coating, and some other variants that are widely used in flexible electronics [123]. Some of them offer the possibility of large-scale flat area layer deposition, which is potentially attractive for the polymer light-shifting layers. However, for technological success, the specific ink density, viscosity and uniformity must be preserved. In addition, the base material (e.g., solar cell or module) must be specially prepared. Some early experiments with the printed light converting layers have been reported, but there is a lack of significant progress so far [124].

## 3. Application in LSC (Luminescent Solar Concentrator) Systems

**Figure 9:**
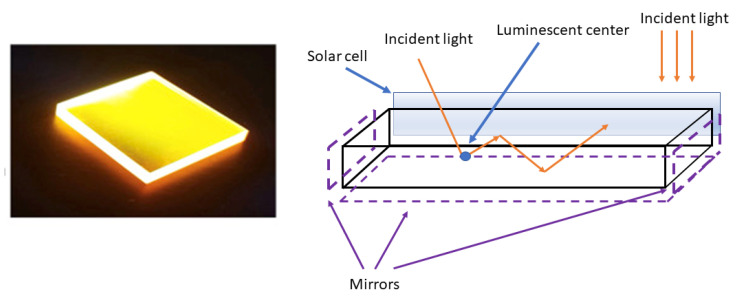
The idea of the LSC cell and the prototype of the luminescence layer with guided light direction and monochromatic emission, based on [126].

The practical application of the luminescence effect in photovoltaics may be achieved in different device configurations. Initially, the most popular method was the edge LSC structure mentioned above. The main idea of this device is rather old [125], and is based on the absorption of re-emitted light on the edge of a large, flat absorbing layer that is enhanced by luminescent agents, which is presented in Figure 9.

In this case, many authors proposed a thin concentrator structure with light trapping and guided light direction [127,128]. The effective advantages of this approach are the large optical absorption area of the luminescent layer and the limited costs, due to the low share of the semiconductor structure in the total module volume.

Unfortunately, the practical realization of this concept brought about several serious problems. Most importantly, the active photovoltaic converter area is only a fraction of the total device dimension, and the adjustment of flat solar cell stripes and their contact systems for this application are very complicated. Moreover, the light trapping system is not efficient enough, since the emission from the top and bottom sides of the concentrator cannot be neglected. The complete flexibility of this system has not been achieved to this point, which eliminates some important advantages of the thin-film photovoltaics. Due to this factor, the typical efficiency gain was normally at the level of 1–20%, with a relatively low overall efficiency of a few percent [129].

**Figure 10:**
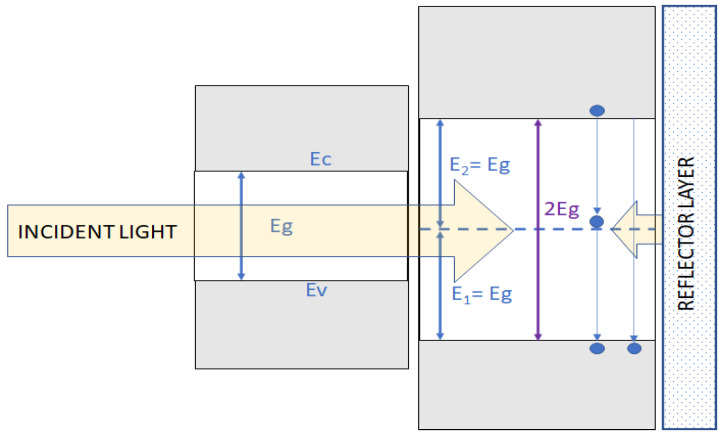
The concept of a down-converting luminescent concentrator with a back light reflector, based on the idea presented in [130].

Another method of luminescent layer construction is to put it in the front or in the rear position directly over the reflection layer, which was first prosed and discussed in detail by Trupke et al. [130]. In this mechanism, the authors postulated the emission of the photons from the luminescent converter layer by the recombination between the valence band and the conductive band, or by the help of the impurity (dopant) level, which is placed in the middle of the material bandgap. The idea of this converter construction is shown in Figure 10.

The authors of this concept calculated the theoretically possible efficiency improvement for such a system, taking into consideration the various band gap of solar cell semiconductor material, with the maximum result of 39.6% for homojunction cells with an Eg = 1.05 eV. This solution may be particularly useful for bifacial solar cells, which are a dynamically growing part of the contemporary PV market [131]. Nevertheless, the practical realization of this idea is still not effective due to several problems connected mostly with the absorption and thermal losses of high-energy photons in a solar cell and the back reflector system’s effectiveness. Thus, there may be greater potential in a front-located converter concept.

**Figure 11:**
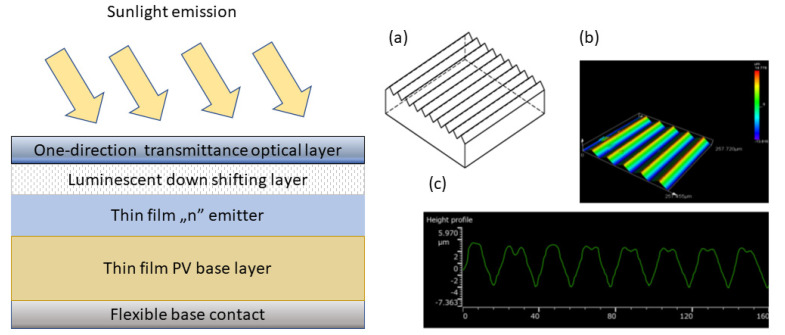
A proposed new structure of a thin film solar cell with a luminescence–based flat optical converter with polymer texturization for antireflective coating; (**a**) polymer foil pattern; (**b**) a photograph of the actual prototype; (**c**) a profile of the foil cross-section with the specific dimensions. Based on [132].

The alternative experimental optical conversion device was proposed as the solution to all of the aforementioned challenges [132]. In this case, the selective transparent layer deposited on top of the down-converting layer is used to increase the efficiency of the device by directing the highest quantity of photons towards the solar cell. Additionally, the specific stack of optical layers for the enhancement of light trapping was proposed. This, combined with the new approach with regard to the application of the selective reflective layer, enables the multiplication of the active device area. The concept and of this device and the idea of polymer antireflecting texturization is schematically explained in Figure 11.

In this approach, the whole area of the solar cell is directly illuminated by the light emitted from the luminescent layer. Additionally, the visible light from the AM 1.5 emission spectrum is also accessible for the PV conversion, contrary to the traditional LSC structure. Furthermore, the flexibility of the complete PV device is completely preserved due to the polymer base of the luminescent layer.

The critical part of this approach is the preparation of the proper luminescent converting layer as well as the functional one-direction transmittance optical layer (the so-called Venetian mirror). Normally, this one-way transmission effect, utilized in many optical applications, is an optical trick that is based on the difference in light intensity on both sides of a mirror, which is not effective from an energy balance point of view. Nevertheless, it appeared that, although difficult, the proper functionalization of the standard PET polymer layer may lead to this effect in the functional form. The results for the optimal configuration (a triangle pyramidal shape with an average 48° tilt angle, illuminated by perpendicular light) in both sides of transmission is shown in Table 3.

**Figure 12:**
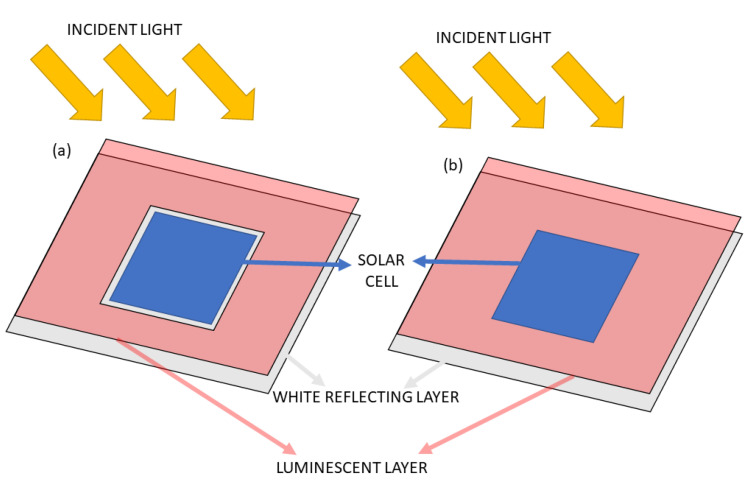
The alternative construction of LSC with a solar cell transparent window; (**a**) and full cover of the module by the luminescent layer (**b**). Based on [78].

An alternative approach to all described methods is the idea of frame LSC construction, where a luminescent layer surrounds the active cell area [133]. This construction is presented schematically in Figure 12. The advantage of such an arrangement is the possibility of using a traditional solar cell instead of an edge cell, a large conversion layer area, low overall costs, and in the variant (a), the strong reduction of the parasitic light absorbance in the luminescent layer.

These issues typically strongly influence the final efficiency gain and may often lead to the complete neglect of the positive light shifting effect. Nevertheless, many interesting and relatively simple construction modifications to the standard silicon modules were presented, which may improve their overall efficiency and extend the potential photoconversion wavelength range.

## 4. Conclusions

The presented article indicates possible development directions and summarizes the most important achievements in this field on the basis of a wide literature search and the technology experience of the author. Throughout the last decades, many materials and practical methods for the adjustment of the incoming solar radiation modifications for solar cells and modules were proposed, but very limited success was achieved in the practical applications. It is clearly visible that the material processing advance, accompanied by the proper layer deposition technology, plays a key role in the preparation of a functional, cheap and effective converting layer. The popularity of this concept is growing, along with the pursuit of the higher conversion efficiency of photovoltaic devices. Additionally, many presented solutions allow for the preservation of a relatively simple production process and a stable, robust module construction. In order to achieve the commercial success of the reviewed concept, some serious issues must be addressed. The first and most important is the construction of an effective polymer matrix, which will not remove the action of the luminescent agents, and will simultaneously transfer the biggest part of the visible insolation to the cell surface. This factor is relatively easy to validate by the evaluation of the solar cell efficiency gain, but difficult with regard to the more precise analysis, and must always be optimized towards specific solar cell parameters. Additionally, luminescence efficiency, the long-term stability of the layer, and an overall production steps arrangement must be optimized toward the high speed and the low cost of the deposition.

The great diversification of the used active agents, matrices, compositions, and module structures provides a wide field for future creativity and promises some commercial-ready products in a predictable future. Amongst them, some materials such as rare earth elements, chalcogenides, and particularly QDs perovskites seem particularly promising. The development of the polymer matrix may be realized on the basis of some highly-transparent and durable materials such as cyclo olefin polymers instead of the traditional PMMA. The deposition process should be based on some inexpensive and scalable technology such as screen printing or spray coating for fast roll-to-roll mass-scale production organization. Nevertheless, many important issues connected with all of these steps should be addressed to obtain satisfactory efficiency improvement and operation time at the level of the currently manufactured PV modules before entering the market.

## Figures and Tables

**Table 1:** Metal oxides NPs summary by type, production technology and achieved parameters [31,32,33,34,35,36,37,38].

Material	Preparation Technology	Obtained Nanoparticle Size
MgO, TiO_2_, Fe_3_O_4_, WO_x_	Solvothermal Synthesis	Single nanometers
ZnO	Physical Vapour Synthesis	8–75 nm
ZnO	Mechanochemical processing	18–40 nm
V_2_O_5_, WO_3_, ZnO, SnO_2_, NiO, ZrO_2_, Cu/Cu_2_O	Pulsed Laser Ablation	20 nm
TiO_2_, ZnO, MgO, CuO, ZrO_2_, SnO_2_	Sol-gel	Bigger than 20 nm
TiO_2_, ZnO, MgO, CuO, ZrO_2_, SnO_2_ Fe_3_O_4_, WO_x_, NiO, Cu/Cu_2_O	Milling	Bigger than 200 nm

**Table 2:** Basic parameters of transparent polymers, based on [109,110].

Parameter	Cyclo Olen Polymer	Silicone Polymers	Acrylic Polymers
Transparency range above 50%	UV-nIR	UV-nIR	UVA-nIR
Refractive index at a wavelength of 430 nm	1.53	1.41	1.49
Maximum operating temperature [°C] (glass point)	110	200	70
Thermal expansion coefficient [1 × 10^−7^/°C]	600	2750	720
Level of UV degradation resistance	Medium	Medium	Low
Young’s modulus [GPa]	2.1	0.002	3
Tensile strength [MPa]	52	11	70

**Table 3:** Total transmittance and reflectance for the triangle texturization pattern, obtained for wavelength λ = 589 nm for PET foil (based on [132]).

Air (n = 1) to PET (n = 1.58) Direction	PET (n = 1.58) to Air (n = 1) Direction
Triangular pattern	Triangular pattern
Absolute transmission = 93%	Absolute transmission = 0%
Absolute reflectance = 7%	Absolute reflectance = 100%

## Data Availability

Not applicable.
